# Occupational exposure to anesthetic agents and mental health among anesthetic technicians

**DOI:** 10.1192/j.eurpsy.2025.1369

**Published:** 2025-08-26

**Authors:** M. A. Ghrab, I. Sellami, A. Haddar, A. Feki, M. Hajjaji, M. L. Masmoudi, K. Jmal Hammami

**Affiliations:** 1 Occupational medicine, University Hospital Hedi Chaker; 2 LR/18/ES-28, University of Sfax; 3 Rheumatology, University Hospital Hedi Chaker, Sfax, Tunisia

## Abstract

**Introduction:**

Exposure to anesthetic agents (AA) has long been a subject of concern, both for patients and healthcare workers. Recent studies highlighted potential associations between a prolonged exposure and healthcare concerns, including psychological effects such as depression.

**Objectives:**

The aim of this study was to assess signs of depression among anesthetic technicians (AT) and evaluate its associated factors.

**Methods:**

We conducted a cross-sectional study among AT in two University Hospitals in Sfax, Tunisia, between January and July 2024 during periodic health assessment visits. Sociodemographic and professional data were collected. The Patient-Health-Questionnaire-9 (PHQ-9) was used to assess signs of depression.

**Results:**

Our population consisted of 60 AT with a mean age of 47.9±7.1 years. Two participants (3.3%) were males. Nine participants (15%) had a known psychiatric history. The mean seniority was 24±7.5 years in healthcare and 10.4±8.1 years in the current ward. The mean duration of exposure to anesthetic gases was 18.3±10.7 years. Sevoflurane was the most utilized AA, used by 75% of the population. Ninety-five percent of the population had shift work.

The median PHQ-9 score was 7 interquartile range IQR [2;11]. Moderate to severe signs of depression were found in 26.7% of the population. Duration of exposure to anesthetic gases was significantly lower among those presenting moderate to severe signs of depression (11 IQR [5;21]) compared to those presenting no or mild signs of depression (20 IQR [11;28]) (p=0.02).

**Conclusions:**

Long professional exposure to AA showed lower depression scores among AT. Comprehensive studies are needed to evaluate the broader mental health and cognitive effects of these agents. Understanding these dynamics is essential for ensuring the safety of healthcare professionals.

**Disclosure of Interest:**

None Declared

